# Supraacetabular fossa is more prevalent in acetabular protrusion than in dysplastic hips

**DOI:** 10.1093/jhps/hnaf054

**Published:** 2025-09-02

**Authors:** Julien Hirt, Vera M Stetzelberger, Alexander F Heimann, Vlad Popa, Joseph M Schwab, Moritz Tannast

**Affiliations:** Department of Orthopaedic Surgery and Traumatology, HFR Fribourg Hospital, University of Fribourg, Fribourg, Switzerland; Department of Orthopaedic Surgery and Traumatology, HFR Fribourg Hospital, University of Fribourg, Fribourg, Switzerland; Department of Orthopaedic Surgery and Traumatology, HFR Fribourg Hospital, University of Fribourg, Fribourg, Switzerland; Department of Orthopaedic Surgery and Traumatology, HFR Fribourg Hospital, University of Fribourg, Fribourg, Switzerland; Department of Orthopaedic Surgery and Traumatology, HFR Fribourg Hospital, University of Fribourg, Fribourg, Switzerland; Department of Orthopaedic Surgery and Traumatology, HFR Fribourg Hospital, University of Fribourg, Fribourg, Switzerland; Department of Orthopaedic Surgery, Inselspital, Bern University Hospital, University of Bern, Bern, Switzerland

## Abstract

The supraacetabular fossa (SAF) is a bony fossa in the acetabular roof’s load-bearing region. Its role in acetabular development and hip morphology remains unclear, with potential implications for hip preservation procedures. We aimed to (i) determine the prevalence of SAF by age, (ii) measure its dimensions in patients with hip pain undergoing magnetic resonance (MR) arthrogram, and (iii) assess associations between SAF and acetabular or femoral morphologies. We performed a retrospective analysis of patients with hip pain who underwent MR arthrograms. SAF were classified as type 1 (unfilled) or type 2 (cartilage-filled), and its dimensions measured. Acetabular and femoral morphology were assessed on radiographs, and hips were categorized into 13 morphology groups. 697 hips from 615 patients were included. SAF was present in 10.9% (76/697) of hips, with affected patients being younger (24.3 ± 7.6 years) than those without SAF (31.8 ± 11.2 years, *P* < 0.001). Type 1 SAF had a mean depth of 3.1 mm *versus* 4.8 mm for type 2 (*P* < 0.001). SAF prevalence was 30.8% in acetabular protrusion and 5.2% in dysplasia (*P* = 0.0135). Acetabular protrusion (OR 3.78, *P* = 0.03) were risk factors for SAF, but no associations were found with femoral morphology. SAF occurs predominantly in patients under 25 years and is nearly six times more common in acetabular protrusion than dysplasia. Future studies should investigate the implications of SAF on acetabular rim trimming and reorientation procedures, particularly focusing on their effect on residual joint contact forces.

## INTRODUCTION

The development of the acetabulum is a complex process, primarily driven by the endochondral ossification of the triradiate cartilage (TRC) [1]. The TRC has a Y-shaped configuration and is located at the junction of the three pelvic bones—the ilium, ischium, and pubis. Growth occurs eccentrically from the intersection point of its three arms [2]. It typically closes around the age of 12 in girls and 14 in boys [3]. Alterations in the TRC have been shown to significantly influence acetabular development, with a premature fusion resulting in dysplastic acetabular morphology [4].

The supraacetabular fossa (SAF) is a bony fossa located in the load-bearing region of the acetabular roof, usually around the 12 o’clock position [5]. SAF are divided in two types: type 1 is an unfilled bony fossa, while type 2 is filled with cartilage [6, 7]. On magnetic resonance (MR) arthrogram a SAF is typically visualized as a well-defined fossa with smooth border, lacking any surrounding bone edema [5]. It is crucial to distinguish SAF from clearly pathologic chondrolabral lesions or acetabular cysts. Furthermore, the size of the SAF might influence the distribution of forces in the weight-bearing zone—relevant in acetabular reorientation or trimming procedures [8].

To date, no study has shown a pathological role for SAF, but it remains unclear whether SAF are merely anatomic variants or if they play a role in acetabular development and, ultimately, influence hip morphology.

Therefore, we asked: (i) What is the prevalence by age, (ii) dimensions of SAF in patients with hip pain undergoing MR arthrogram? and (3) are SAF associated with specific acetabular or femoral morphology?

## MATERIALS AND METHODS

### Study design and settings

After local institutional review board approval, we performed a retrospective, single center, diagnostic study.

### Patients

**Figure 1:**
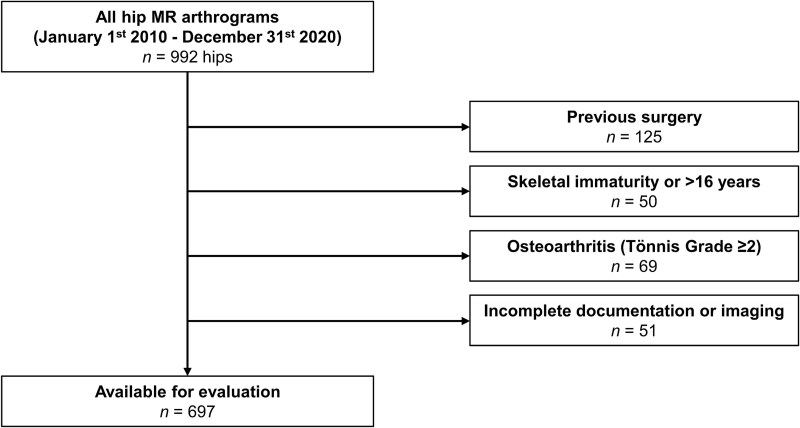
Inclusion and exclusion criteria are shown.

We selected all patients with symptomatic hip pain who presented at our institution between January 2010 and December 2020. Inclusion criteria was an available MR arthrogram of the hip. Exclusion criteria were patients aged < 16 to ensure closure of the TRC [1] (*n* = 50), Tönnis Grade ≥ 2 osteoarthritis (*n* = 69), hips with previous surgery (*n* = 125), and cases with incomplete clinical documentation or incomplete/uninterpretable imaging (*n* = 51). Out of the 992 hips initially reviewed, 697 (70.3%) were included in the analysis (Fig. 1).

MR arthrograms were performed according to our institutional standardized protocol. Details have been previously published [9]. In addition to MR arthrograms, anteroposterior (AP) pelvis radiograph and an axial cross-table view of the proximal femur, using a standard technique, were available for every patient [10]. Briefly, the film-focus distance was 1.2 m and the legs were internally rotated ~15° to compensate for femoral antetorsion. For the AP view, the central beam was directed to the midpoint of the superior border of the symphysis and the center of a line connecting both anterosuperior iliac spines. For the axial view, the central beam was directed to the inguinal fold, while the film was close to the iliac crest and oriented at 45° to the sagittal plane.

### SAF classification and measurement

**Figure 2:**
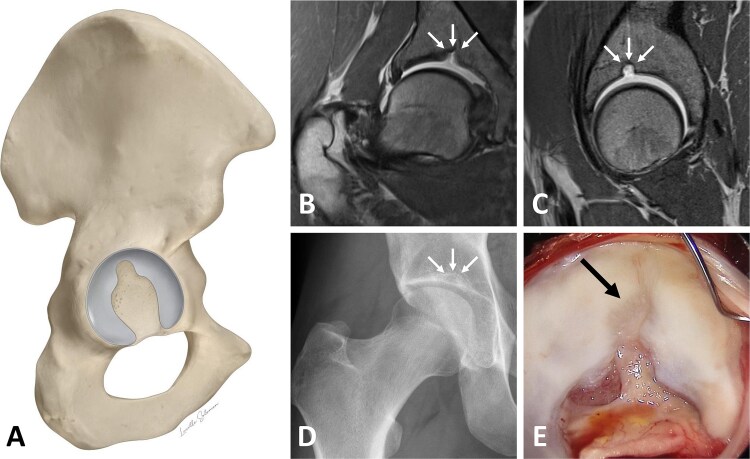
A-E illustration of a supraacetabular fossa (SAF) type 1, which contains no cartilage. (A) Representation of the hemipelvis with view on the acetabulum. Coronal (B) and sagittal (C) MR arthrogram views of the SAF, which appears hyperintense because it is filled with contrast material. (D) Anteroposterior radiograph showing the SAF. (E) Intraoperative view of the acetabulum during a surgical hip dislocation procedure.

**Figure 3:**
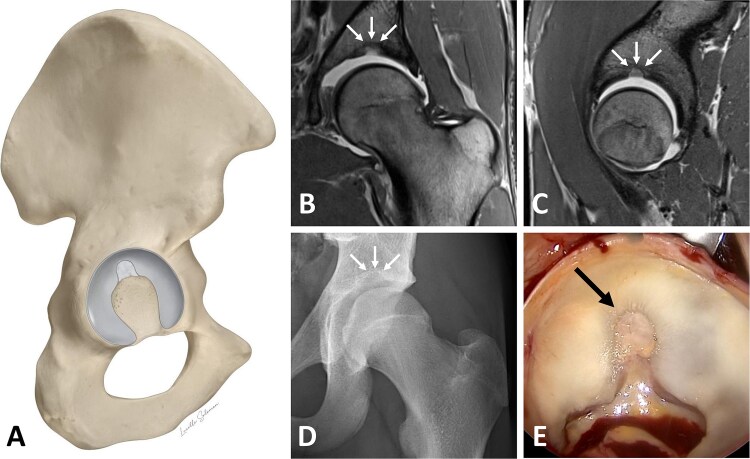
**(**A-E) Illustration of a supraacetabular fossa (SAF) type 2, which is filled with cartilage. (A) Representation of the hemipelvis with view on the acetabulum. Coronal (B) and sagittal (C) MR arthrogram views of the SAF. Due to the presence of cartilage, no contrast agent can enter the fossa, resulting in it not appearing hyperintense. (D) Anteroposterior radiograph showing the SAF. (E) Intraoperative view on the SAF during a surgical hip dislocation procedure, revealing the cartilage inside the SAF.

**Figure 4:**
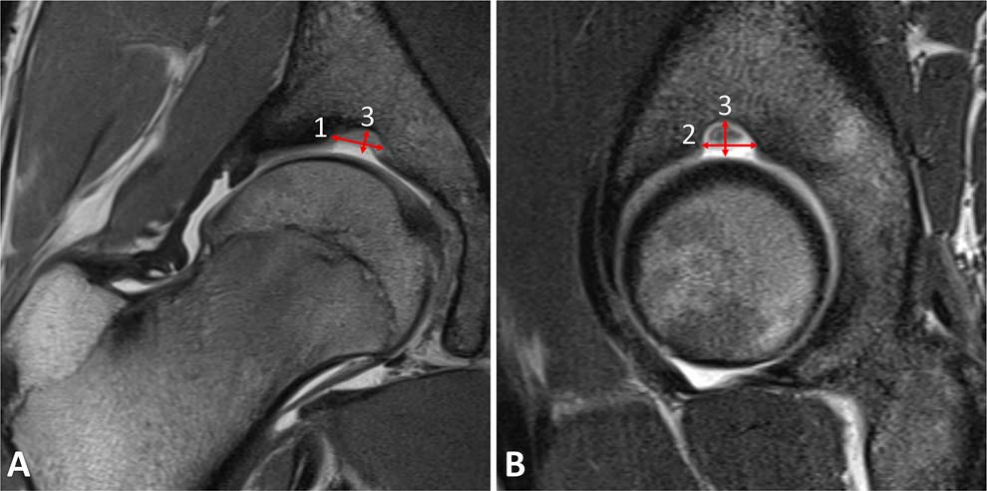
**(**A-B) Measures of SAF were made on coronal (A) and sagittal (B) planes. The mediolateral width (i) was measured on coronal sequences, and the anteroposterior width (ii) on sagittal sequences. The craniocaudal width (3) was measured on both sequences and the higher value was selected.

SAF were identified on MR arthrogram and classified into two types: SAF type 1 produces a hyperintense signal on MR arthrography because it contains little or no cartilage and is therefore filled with contrast material (Fig. 2). SAF type 2 does not produce a hyperintense signal because it is filled with cartilage and therefore does not allow the contrast material to enter (Fig. 3). SAF were then measured, according to the technique described by Dietrich *et al* [7]: mediolateral width was measured on coronal sequences, and AP width on sagittal sequences. Craniocaudal depth was measured on both sequences and the higher value was used (Fig. 4).

### Acetabular and femoral morphology

Radiographic parameters were evaluated using previously validated software (Hip2Norm [11, 12]; University of Bern, Bern, Switzerland). Based on AP pelvis radiographs, the software performs a three-dimensional analysis of the hip morphology and measures commonly used radiographic parameters. Femoral torsion was directly measured on MR arthrograms, according to the technique described by Murphy [13, 14].

Using these analyses, we divided the study population into 13 groups based on different acetabular and femoral morphological features. Each hip was classified according to acetabular coverage as dysplasia (lateral center edge (LCE) < 22°), normal coverage (LCE 22°–34°), overcoverage (LCE > 34°–39°), severe overcoverage (LCE > 39°), or acetabular protrusion (femoral head touches or crosses the ilioischial line). Additionally, hips were classified in the retroversion group if they had a retroversion index > 30%, a positive cross-over sign, a positive posterior wall sign, and a positive ischial spine sign [15]. Femoral neck-shaft morphology was categorized as coxa vara (caput-collum-diaphyseal angle (CCD) ≤ 125°), coxa ‘normal’ (CCD 125°–140°) or coxa valga (CCD ≥140). Based on femoral torsion, hips were classified as having reduced femoral torsion (<10°, Murphy method [14]), normal femoral torsion (10°–25°), increased femoral torsion (>25°). In addition, hips were categorized as having acetabular retroversion or cam morphology (alpha angle > 55°) if they met the respective criteria. Hips with Legg-Calvé-Perthes disease were classified separately. Assignments were made according to previously published definitions, as listed in Table 1. According to this procedure, a hip could be classified in one or more groups.

**Table 1:** Radiographic definitions for acetabular and femoral morphologies.

**Groups**	**Definition**
Acetabular morphologies	
Dysplasia	LCE < 22° [16]
Normal coverage	LCE 22°–34° [16]
Overcoverage	LCE > 34°–39° [16]
Severe overcoverage	LCE > 39° [16]
Acetabular protrusion	Femoral head touches or crosses the ilioischial line [10]
Acetabular retroversion	Retroversion index > 30%, positive cross-over sign, positive posterior wall sign, and positive ischial spine sign [15]
Femoral morphologies	
Reduced torsion	Torsion < 10° [17]
Normal torsion	Torsion 10°–25° [17]
Increased torsion	Torsion > 25° [17]
Coxa vara	CCD ≤ 125° [18]
Coxa ‘normal’	CCD > 125°—<140° [18]
Coxa valga	CCD ≥140° [18]
Cam morphology	Alpha angle > 55° [19]

CCD: caput-collum-diaphyseal; LCE: lateral center edge

Demographic data including sex, laterality, BMI, and age at MR arthrogram were recorded and compared between hips with and without SAF.

### Statistical analysis

We tested normal distribution with the Shapiro–Wilk test. Differences between groups were assessed with the Student t-test for data that followed a normal distribution. For non-normally distributed data, the Mann–Whitney U test was used. Categorical data were compared with the chi-square test. To examine associations between acetabular and femoral morphologies and the presence of SAF, we performed a univariate logistic regression analysis. Intrarater and interrater reliability were calculated using the intraclass correlation coefficient (ICC). Based on the ICC values, intrarater and interrater reliability were categorized as follows: poor (<0.21), fair (0.21 to 0.40), moderate (<0.40 to 0.60), good (>0.60 to 0.80), or excellent (>0.80 to 1.00) [20]. Statistical analysis was performed using R (R Foundation, Vienna, Austria).

### Intrarater and interrater reliability

Fifty randomly selected hips were independently reviewed by three orthopaedic residents to assess the presence of SAF. SAF were classified either as type 1 or type 2 and measured on sagittal and coronal sequences. The identification and measurements of SAF were conducted twice at two separate times. Excellent intrarater reliability was found for the identification of the SAF among the three observers (Table 2). Measures of the SAF showed an excellent intrarater reliability for all measurements except for the craniocaudal width in one observer, which showed a good reliability (ICC = 0.75; 95% CI, 0.57–0.86). Overall, the interrater reliability was good for the detection of SAF as well as for SAF measurements. The highest score was found for the identification of SAF (ICC = 0.79; 95% CI, 0.66–0.87), while the lowest score was found for the measurement of the craniocaudal width (ICC = 0.73; 95% CI, (0.58–0.84).

**Table 2:** Results of the intrarater and interrater reliability for the identification of the supraacetabular fossa and their measurements.

	**ICC intrarater**			**ICC interrater**
	**Observer 1**	**Observer 2**	**Observer 3**	
Identification of SAF	1.00	0.97 (0.94–0.98)	0.93 (0.87–0.96)	0.79 (0.66–0.87)
Anteroposterior width	0.99 (0.99–0.997)	0.96 (0.94–0.98)	0.97 (0.95–0.98)	0.75 (0.60–0.85)
Mediolateral width	0.97 (0.95–0.98)	0.89 (0.80–0.94)	0.93 (0.88–0.96)	0.74 (0.58–0.84)
Craniocaudal width	0.99 (0.99–1.00)	0.98 (0.96–0.99)	0.75 (0.57–0.86)	0.73 (0.58–0.84)

Values are given as intraclass coefficients with 95% confidence interval in parentheses. ICC: intraclass coefficient. SAF: supraacetabular fossa.

## RESULTS

### Age-specific prevalence and dimension of SAF

A total of 697 hips from 615 patients were included in the study, with 13.3% (82/615) patients undergoing bilateral hip imaging. In our study there were 42.9% (264/615) males and 51.4% (358/697) right hips. SAF was identified in 10.9% (76/697) of hips (Table 3). The mean age of patients with SAF was 24.3 years (±7.6) *versus* a mean age of 31.8 years (±11.2) in patients without SAF (*P* < 0.001). Mean BMI in patients with SAF was 22.9 (±3.3) *versus* 25.0 (±4.7) in patients without SAF (*P* < 0.001). There were 20 (26.3%) type 1 and 56 (73.7%) type 2 SAF. In the patients who underwent bilateral imaging, 21 (12.8%) SAF were identified. Five (31.3%) patients had bilateral SAF (*n* = 10), and 11 (68.8%) had unilateral SAF. One patient had bilateral SAF type 1, two patients had bilateral SAF type 2, and two patients had SAF type 1 on one side and SAF type 2 on the other side.

**Table 3:** Demographic parameters and dimensions of the supraacetabular fossa.

**Parameters**	**SAF type 1**	**SAF type 2**	***P* value** **SAF type 1 versus SAF type 2**	**Hips without SAF**	***P* value** **SAF versus without SAF**
Demographic features					
Number of SAF	20	56	N/A	621	N/A
Age at MRA (years)	21.7 ± 5.5 (16.2–35.1)	25.3 ± 8.1 (16.4–48.0)	**0.03087**	31.8 ± 11.2 (16.0–68.3)	**<0.001**
BMI, kg/m^2^	22.9 ± 2.2 (19.3–26.3)	22.9 ± 3.6 (18.3–31.6)	0.545	25.0 ± 4.7 (16.3–47.5)	**<0.001**
Sex (% men)	10 (50.0)	19 (33.9)	0.3164	269 (43.3)	0.4621
Side (% right)	9 (45.0)	33 (58.9)	0.416	316 (50.9)	0.5491
Dimensions of SAF					
Anteroposterior width (mm)	6.8 ± 3.2 (1.4–15.6)	7.3 ± 2.9 (1.8–14.9)	0.4681	N/A	N/A
Mediolateral width (mm)	6.0 ± 2.4 (2.5–12.5)	6.5 ± 3.5 (1.8–15.5)	0.7278	N/A	N/A
Craniocaudal width (mm)	3.1 ± 2.3 (1.4–11.7)	4.8 ± 2.8 (1.4–15.5)	**<0.001**	N/A	N/A

Continuous values are expressed as mean ± SD (range); other values are presented as number with percentage in parenthesis unless noted otherwise. SAF: supraacetabular fossa; MRA: magnetic resonance arthrogram; BMI: body mass index; N/A: not available. Bold values indicate statistically significant differences (*p* < 0.05).

**Figure 5:**
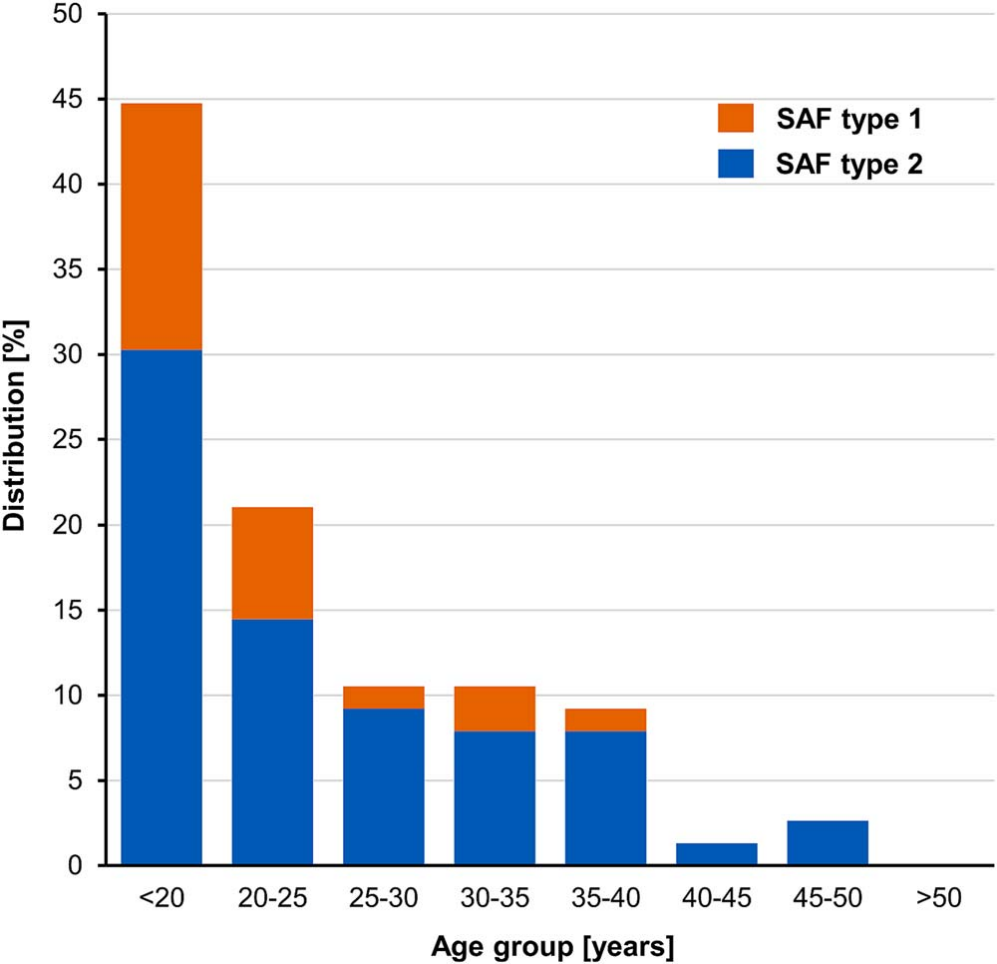
The distribution of the supraacetabular fossa (SAF) shows that almost 45% of all SAF were identified in patients under 20 years old.

By age group, 44.7% (34/76) of all SAF were identified in patients younger than 20 years, 21.1% (16/76) in patients aged 20–25 years, 10.5% (8/76) in patients aged 25–30 years, 10.5% (8/76) in patients aged 30–35 years, 9.2% (7/76) in patients aged 35–40 years, and 3.9% (3/76) in patients older than 40 years (Fig. 5). No SAF cases were identified in patients over 50 years. The mean age in patients with SAF type 1 was 21.7 years (±5.5) compared to a mean age of 25.3 years (±7.6) in patients with SAF type 2 (*P* = 0.03087).

Mean craniocaudal depth for SAF type 1 was 3.1 mm (± 2.3) *versus* 4.8 mm (± 2.8) for SAF type 2 (*P* < 0.001; Table 3).

### Association of SAF with specific acetabular or femoral morphology

**Figure 6:**
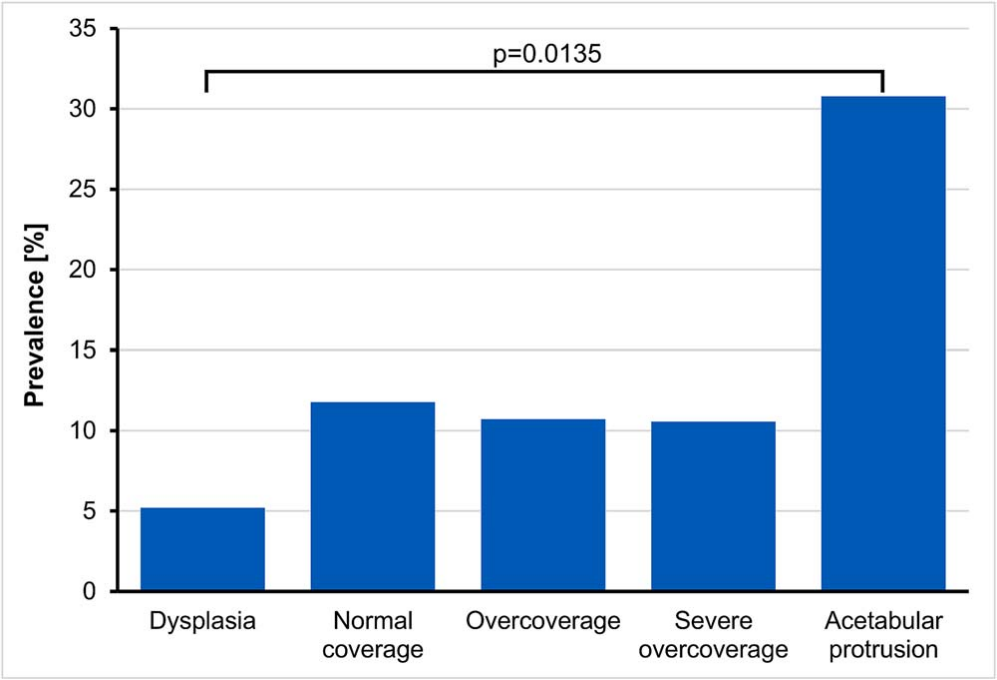
Prevalence of the supraacetabular fossa increases with the increase in acetabular coverage.

When assessing by acetabular coverage, the prevalence of SAF was 30.8% (4/13) in hips with acetabular protrusion and 5.2% in hips with dysplasia (4/77, *P* = 0.0135, Fig. 6, Table 4). When assessing by femoral torsion, the prevalence of SAF was 12.7% (31/244) in hips with increased femoral torsion and 6.4% (6/94) in hips with reduced femoral torsion (*P* = 0.141). Regarding femoral neck-shaft morphology, SAF was identified in 8.8% (12/136) of hips with coxa vara and 9.6% (5/52) with coxa valga (*P* = 1.0). The prevalence of SAF in hips with cam morphology was 11.3% (51/450), compared to 10.4% (21/201) in hips without cam (*P* = 0.843).

**Table 4:** Prevalence of hips with a supraacetabular fossa according to acetabular or femoral morphologies.

**Groups**	**Prevalence of SAF**	***P* value** *	**Prevalence of SAF type I among SAF**	**Prevalence of SAF type II among SAF**
**Acetabular morphologies**				
Normal coverage	11.8% (35/297)		25.7% (9/35)	74.3% (26/35)
Dysplasia	5.2% (4/77)	0.140	75% (3/4)	25% (1/4)
Overcoverage	10.7% (18/168)	0.844	22.2% (4/18)	77.8% (14/18)
Severe overcoverage	10.6% (15/142)	0.829	26.7% (4/15)	73.3% (11/15)
Acetabular protrusion	30.8% (4/13)	0.1381	0% (0/4)	100% (4/4)
Acetabular retroversion	8.5% (13/153)		15.4% (2/13)	84.6% (11/13)
**Femoral morphologies**				
Normal torsion	12.2% (32/263)		21.9% (7/32)	78.1% (25/32)
Reduced torsion	6.4% (6/94)	0.172	16.7% (1/6)	83.3% (5/6)
Increased torsion	12.7% (31/244)	0.961	32.3% (10/31)	67.7% (21/31)
Coxa ‘normal’	11.6% (59/509)		23.7% (14/59)	76.3% (45/59)
Coxa vara	8.8% (12/136)	0.446	33.3% (4/12)	76.7% (8/12)
Coxa valga	9.6% (5/52)	0.843	40% (2/5)	60% (3/5)
Absence of cam	10.4% (21/201)		38.1% (8/21)	61.9% (13/21)
Cam morphology	11.3% (51/450)	0.843	21.6% (11/51)	78.4% (40/51)
Legg-Calvé-Perthes	9.5% (2/21)		50% (1/2)	50% (1/2)

^*^ *P* value compared to normal morphological group; SAF: supraacetabular fossa.

Univariate logistic regression analysis identified acetabular protrusion (OR 3.78, 95% CI 1.00–11.9, *P* = 0.03) as relative risk factors for SAF (Table 5). We observed no relative risk for SAF in femoral torsional morphologic groups or femoral neck-shaft morphologic groups.

**Table 5:** Results of the univariate logistic regression models examining associations between morphological groups and the presence of SAF.

**Groups**	**OR**	**95% CI**	***P*-value**
**Acetabular coverage**			
Dysplasia	0.42	0.12, 1.04	0.1
Normal coverage	1.17	0.72, 1.89	0.5
Overcoverage	0.97	0.54, 1.67	>0.9
Severe overcoverage	0.96	0.51, 1.70	0.9
Acetabular protrusion	3.78	1.00, 11.9	**0.03**
Retroversion	0.71	0.36, 1.29	0.3
**Femoral torsion**			
Reduced torsion	0.48	0.18, 1.06	0.1
Normal torsion	1.13	0.68, 1.86	0.6
Increased torsion	1.22	0.73, 2.02	0.4
**Neck-shaft morphology**			
Coxa vara	0.75	0.38, 1.39	0.4
Coxa ‘normal’	1.32	0.76, 2.39	0.3
Coxa valga	0.86	0.29, 2.04	0.8
**Head–neck morphology**			
Cam	1.10	0.65, 1.91	0.7
Legg-Calvé-Perthes	0.76	0.12, 2.70	0.7

OR: Odds Ratio, Cl: Confidence Interval. Bold values indicate statistically significant differences (*p* < 0.05).

## DISCUSSION

The aim of our study was to determine the prevalence by age and morphological associations of SAF in candidates for hip preserving procedures. We identified a similar prevalence rate of SAF in patients with hip pain to those that have been reported in the literature [6, 7]. In addition, we identified a substantially higher prevalence rate of bilateral SAF in those who underwent bilateral hip imaging.

Our findings indicate an age-dependent prevalence of SAF in patients with symptomatic hip pain. Patients with SAF were younger than those without, and a majority of SAF cases occurred in patients under 20 years of age. For SAF type 1 specifically, more than half were identified in individuals under 20 years, with only three cases observed in patients older than 30: one 31-year-old male with bilateral SAF type 1 and one 35-year-old male with unilateral SAF type 1. Our observations align with those of Vaeth *et al.*, who reported a SAF prevalence of 35.6% in a cohort aged 4–25 years [21]. They also observed that SAF type 1 was more frequently found in younger patients (median age 16 years), while the median age for SAF type 2 was 21 years. Vaeth *et al.* hypothesized that SAF type 1 may represent a developmental variant of the acetabulum that may fill with cartilage (SAF type 2) and eventually obliterates with age [21].

The presence of SAF appears to be related to acetabular coverage. Hips with acetabular protrusion showed a six-fold increase in prevalence compared to dysplastic hips. Acetabular protrusion is a rare entity what reflects in the small number of cases in this study. While the association was significant, the small sample size may amplify the impact of individual cases on the calculated prevalence.

Alterations in the TRC have been shown to significantly influence acetabular development [4]. An experimental study on rabbits demonstrated that premature fusion of the TRC can lead to acetabular shallowing and femoral head subluxation [22]. Similarly, children with traumatic TRC injuries are at higher risk of developing acetabular dysplasia with subsequent femoral head subluxation [2, 4]. Although the position of the SAF does not exactly correspond to the typical location of the TRC, little is known about potential variations in the orientation of the three arms of the TRC. Our study was not designed to explore the relationship between TRC and SAF. However, our findings indicate a potential link between growth disturbances and the ultimate morphology of the hip. Further research is needed to determine this relationship, as well as identify any influence on acetabular coverage.

Protrusion hips experience lower contact stress compared to dysplastic hips, due to the larger lunate surface and the constrained medial position of the femoral head. In contrast, dysplastic hips tend to exhibit lateral decentration of the femoral head [23]. One could speculate that the reduced contact stress in the weight-bearing zone of protrusion hips may delay the closure of the SAF, while the increased loading seen in dysplastic hips may accelerate its ossification.

Additionally, hips with acetabular protrusion are at a higher risk of failure after open circumferential acetabular trimming performed *via* surgical hip dislocation, with a 10-year survivorship rate of 51% compared to 83% in controls with pincer-type FAI [24]. This raises the question whether outcomes of surgical intervention on these hips may be influenced by the presence of SAF, and this also requires further investigation.

In contrast to the acetabulum, we found no association between the presence of a SAF and femoral torsion or femoral neck-shaft morphology.

Other anatomical variants of the acetabulum have been described [5, 25]. Among them, the stellate crease is a distinct structure located more medially than the SAF [5]. It is devoid of cartilage and has been hypothesized to play a role in synovial fluid circulation by facilitating the flow of synovial fluid toward the articular surface [26].

### Limitations

This study has several limitations. First, the cohort studied consisted exclusively of patients with symptomatic hip pain undergoing MR arthrograms. Consequently, it was not possible to determine the prevalence of SAF in the general population, which may differ in asymptomatic individuals of similar age as bony morphology in symptomatic patients often differ. Additionally, the study was not designed to assess whether the presence of SAF correlates with hip pain.

Second, the study design allowed individual patients to be categorized into multiple morphological groups. While this increased the number of patients in each category, it also introduced potential confounding factors. Additionally, the morphological groups were unequal in size, with some categories including relatively few patients. This is particularly true for the rare entity of acetabular protrusion, which may explain the wide confidence interval. Despite these limitations, this study provides insights into potential correlations and serves as a basis for future research.

Third, not all patients underwent bilateral imaging, as this is not routinely performed in our clinical practice. This limits the ability to evaluate the laterality of SAF and may result in an underestimation of its true prevalence or an inaccurate assessment of bilateral SAF prevalence.

Fourth, the study was not designed to determine whether SAF represents a developmental remnant of the TRC or to assess any potential association between the two. Further research is required to elucidate the origins of SAF and its potential relationship with TRC.

Fifth, our study population predominantly consisted of younger patients, as MR arthrograms are more commonly performed in the context of hip preservation procedures rather than for advanced osteoarthritis. As a result, older adults were underrepresented, which limits our ability to assess the prevalence of SAF in this age group. Finally, measurement of femoral torsion was not possible in 96 cases (13.8%) because the femoral condyles were not included in the MR arthrogram protocol for these patients. This may have affected the distribution of observed SAF in our different morphological groups.

## CONCLUSION

Supraacetabular fossae are found in ~11% of patients with hip pain, predominantly in individuals under the age of 25. While SAF are rare in cases of dysplasia, they are present in up to one-third of hips with acetabular protrusion. Future research should be focused on how the presence of SAF affects outcomes in hip preserving surgeries such as focal and circumferential acetabular rim trimming and periacetabular osteotomy.

## Data Availability

The data underlying this article will be shared on reasonable request to the corresponding author.
